# A systematic review of economic evaluations of enzyme replacement therapy in Lysosomal storage diseases

**DOI:** 10.1186/s12962-022-00369-w

**Published:** 2022-09-19

**Authors:** Eleni Ioanna Katsigianni, Panagiotis Petrou

**Affiliations:** Pharmacy School, Department of Life &Health Sciences, School of Sciences and Engineering, University of Nicosia, 2417 Nicosia, Cyprus

**Keywords:** Cost-effectiveness, Enzyme replacement therapy (ERT), Gaucher disease, Fabry disease, Pompe disease, Lysosomal acid lipase (LAL) deficiency, Lysosomal storage diseases (LSD), Cost, Quality of life

## Abstract

**Objective:**

The objective of this paper is to assess the economic profile of enzyme replacement therapy (ERT) to symptomatic patients with Pompe, Fabry, Gaucher disease and Lysosomal acid lipase (LAL) deficiency.

**Methods:**

A systematic search was performed to retrieve and critically assess economic evaluations of enzyme replacement therapy. Publications were screened according to predefined criteria and evaluated according to the Quality of Economic Studies. Data were narratively synthesized.

**Results:**

The Incremental Cost-Effectiveness Ratio greatly exceeded willingness to pay thresholds. The cost of the medication dominated the sensitivity analysis. For Infantile-onset Pompe’s disease, the incremental cost-effectiveness ratio (ICER) was estimated at €1.043.868 per Quality-adjusted life year (QALY) based on the dose of alglucosidase 40 mg/kg/ week, and €286.114 per QALY for 20 mg of alglucosidase/kg/2 weeks. For adults patients presenting with Pompe disease the reported was ICER € 1.8 million/ QALY. In the case of Fabry disease, the ICER per QALY amounts to 6.1 million Euros/QALY. Respectively for Gaucher’s disease, the ICER /QALY was estimated at € 884,994 per QALY. Finally, for patients presenting LAL deficiency NCPE perpetuated an ICER of €2,701,000/QALY.

**Discussion:**

ERT comprise a promising treatment modality for orphan diseases; nevertheless, it is interlaced with a substantial economic burden. Moreover, the available data on the cost-effectiveness ratio are scarce. For certain diseases, such as Fabry, a thorough selection of patients could exert a beneficial effect on the reported ICER. Steep price reductions are imperative for these products, in the conventional reimbursement pathway or a new assessment framework should be elaborated, which in principle, should target uncertainty.

## Introduction

Lysosomal storage diseases (LSD) describe a group of approximately 50 rare and heterogenous inherited metabolic disorders which are caused by defects in lysosomal function, in the form of loss or deficiency of a specific enzyme [1]. Among them, the most common are Gaucher, Pompe, Fabry, Mucopolysaccharidosis type I (MPS), and Lysosomal acid lipase (LAL) deficiency.

Gaucher disease is the most common lysosomal disease. It is caused by a deficiency of the enzyme lysosomal glucocerebrosidase (GBA1). This leads to accumulation of glucocerebrosidase (GC) in these organs. Gaucher occurs in three types: Type 1 is the most frequent (non-neuropathic, 1: 40,000 to 1: 60,000) and type 3, the chronic, the least common type of disease with prevalence 1: 100,000). Fewer than 10,000 people worldwide are affected by the disease. Imigluerase, a recombinant enzyme modified to enhance its uptake into lysosomes, is used to replace the defective enzyme and it is indicated for symptomatic type 1 and type 3 patients [2]. None of the Enzyme replacement Therapy (ERT)s agents, are indicated for GD2 as treatment has no impact on the rapid progression of its severe neurological symptoms [3]. The option of using ERT to treat Type 2 GD patients has been a controversial issue because of its cost and inconvenience that may only prolong suffering, although in recent years there have been infants that started on therapy [4]. In any case the variable pattern of people presenting with disease and its severity as well as its uncertain outcome makes it difficult to decide whether to initiate ERT. In addition, it is reasonable to consider therapy for Type 2 GD patients in situations where it could be palliative, for example if reduction of organomegaly would alleviate pain, avoid surgery, or facilitate other necessary interventions such as gastric tube placement. The prognosis for type 1 is good, however the survival for type 2 patients is less than two years while for type 3, life expectancy is slightly higher than type 2.

Pompe disease is a progressive metabolic neuromuscular disorder caused by a deficiency of α-acid glucosidase (GAA), an enzyme that breaks down glycogen, due to a mutation in the gene encoding GAA. It affects 1:40.000 births worldwide [5], while the prevalence of classic-infantile Pompe disease is even lower (1:138.000 births [6]). This enzyme deficiency leads to the accumulation of toxic levels of glycogen in cells, thus impeding their normal functions. There are several phenotypes due to the variability that occurs on the age of onset and degree of organ involvement. These subtypes are infantile (IOPD), late infantile, childhood, juvenile, and late onset form (LOPD) [7]. Respiratory failure is the leading cause of morbidity and mortality. Depending on the rate of disease progression, death may occur from early childhood to adulthood [7]. The recombinant human aglucosidase currently comprises the only therapeutic modality [8]. The life expectancy for children who receive supportive care only is poor and it is associated with a 92% mortality rate during the first year of life. The administration of aglucoside has greatly reduced the risk of death by 99% (0.01 hazard ratio; 0.00–0.10 95% CI; p < 0.0001) in patients aged ≤ 6 months and 71% (hazard ratio: 0.29; 0.11–0.81 95% CI; p < 0.018) for patients aged > 6 months to ≤ 36 months according to Castro- Jaramillo [5]. Despite the advances, the mortality in IOPD is still substantial, affecting 28–43% of the individuals.

Fabry disease is an x-linked inherited multisystem lysosomal disorder that leads to insufficient activity of the lysosomal enzyme α-galactosidase due to abnormalities of the responsible GLA gene, which controls its synthesis. This cascades to an incomplete catabolism of glycosphingolipids with α-galactose terminal molecules and their progressive intracellular accumulation with a simultaneous increase in their levels in the blood. The estimated birth prevalence is around 1:40.000 [9]. Symptoms may appear in infancy, childhood, or in rare cases in adulthood where they are less severe. Over time, the disease leads to a progressive renal dysfunction, cardiomyopathy, and stroke leading to significant morbidity and mortality [10]. The treatment aims to improve the patient's quality of life and prevent life-threatening symptoms. ERT gives patients a form of α-galactosidase to reduce symptoms [10]. Α-agalsidase and β-agalsidase are recombinant enzymes, produced in genetically modified human cell line and genetically modified Chinese hamster ovary cells, respectively. They are administered intravenously to replace the defective enzyme and are indicated for symptomatic Fabry disease [2].

LAL deficiency is a rare autosomal recessive inherited metabolic disorder, caused by defects of the LIPA gene, and can lead to the accumulation of cholesteryl esters and triglycerides in various tissues. LAL deficiency spirals to two rare conditions. The early onset Wolman disease (WD) is a rapidly progressive condition whereas in paediatric adult patient (Cholesterol, ester storage disease-CESD) the disease progression is less rapid [11]. The disease severity is related to the presence of residual LAL enzyme activity and indicated by age of onset. LAL deficiency has an estimated prevalence ranging from 1:40,000 to 1:300,000. Life expectancy for severe cases is < 4 months (untreated), while for some attenuated cases (untreated) lifespan may be determined by co-morbidities such as liver failure [11].

Among the array of therapeutic modalities, which have been put forward, ERT has emerged as the mainstay in the treatment of LSD [12]. The context of ERT was first introduced in 1964, however three decades elapsed until a pharmaceutical product entered the market. ERT compensates the underlying enzyme deficiency and the prevention of the symptoms of diseases, which left untreated, could have caused permanent and multifaceted damage to patients and premature death as well.

The financial burden of these conditions is substantial as with any given orphan disease, and the cost of new therapeutic agents is burgeoning. ERT is no exception to this rule, and it is intertwined with soaring costs to the health care system. However, as with any given orphan disease, social equity and social cohesion should be demonstrated and access to orphan drugs comprises a pillar of such policies. So far, little attention has been paid to the economic outcomes of ERT, with the literature primarily focusing on the humanistic and clinical aspects of these conditions. Therefore, in the present review has attempted to bridge this gap. In the context of decision-making in health politics, the notion of economic evaluations has been established as the pinnacle in decision-making in health since the cost of these agents constitutes a fiscal stress test for Healthcare Systems worldwide. To this direction, economic evaluations can bring about significant insights and elucidate the full potentials of each product, capitalizing on all aspects, medical, social, and economic. While cost-effectiveness for individual LSD were published, no systematic reviews of economic evaluations for the entire spectrum of LSD have been performed. Therefore, the aim of our work was to present a holistic perspective of the pharmacoeconomic aspects of ERT in LSD.

## Systematic literature review

**Fig. 1:**
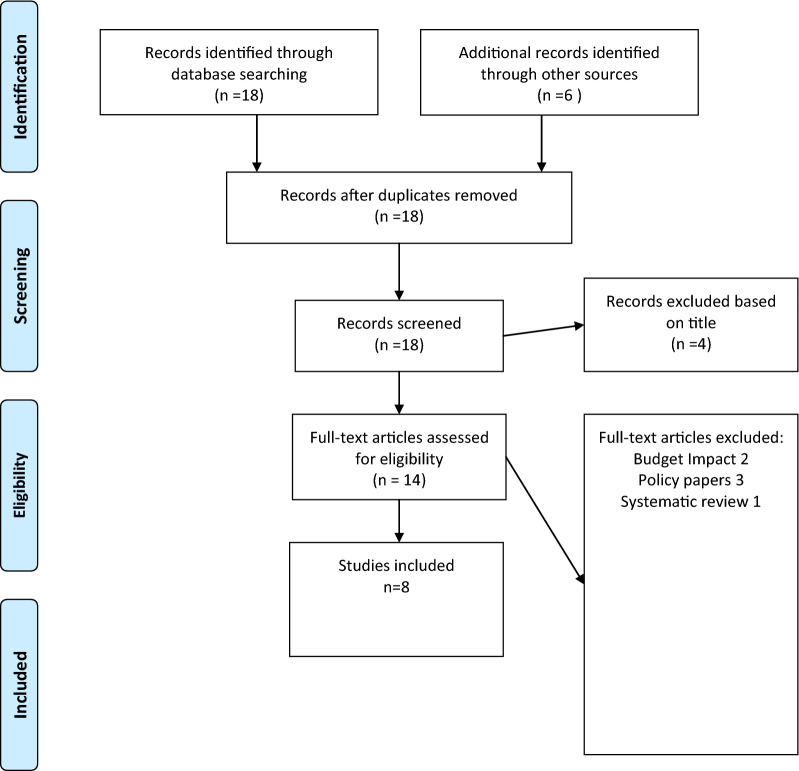
Flow Diagram of literature review

We performed a systematic literature review, which was based on the following PICO (population, intervention, control, outcome) elements and abide by the PRISMA guidelines [13] [Fig. 1.]:

1. Population: patients presenting with Lysosomal storage diseases Pompe, Fabry, Gaucher disease, LAL deficiency and MPS disease
2. Intervention: enzyme replacement therapy
3. Control: standard Care
4. Outcomes: incremental cost-effectiveness ratio (ICER), incremental cost-utility ratio (ICUR)

Search was performed in Cochrane Central Register of Controlled Trials, Cochrane Database of Systematic Reviews, EMBASE, EMBASE Alert, Health Technology Assessment Database, MEDLINE, Scopus, NHS Economic Evaluation Database and Health Technology Assessment (HTA) until 1/3/2022 No restrictions were applied to the publication date or language during the search. The following Mesh terms were used: ((((enzyme replacement therapy (MeSH Terms]) OR (therapies, enzyme replacement [MeSH Terms])) OR (enzyme replacement therapies [MeSH Terms])) AND (analyses, cost benefit [MeSH Terms])) AND (cost benefit analyses [MeSH Terms]). We excluded letters, editorials, correspondences, or comments. The literature search yielded 154 results. Manual search and snow-ball search yielded three more results. Based on title, we excluded 129 studies. Further review led to removal of 19 studies (Fig. 1.). Studies with ceredase were excluded due to withdrawn of the product from the market.

## Results

Among the retrieved articles, three articles referred to Pompe disease. Due to the significant differences in patient characteristics and the consequent financial burden between the onset of infantile Pompe disease (IOPD) and the late onset Pompe disease (LOPD), these two phenotypes are examined separately. Two of the publications included patients with IOPD only, in England/Colombia [5] and the Netherlands [6]. One publication referred exclusively to patients with LOPD in the Netherlands [14]. 14 studies of Fabry disease were identified. Among them, one article was selected for analysis for Fabry disease [9]. which compared the cost-effectiveness of ERT with standard medical care in the Netherlands. One study, by van Dussen et al. [15], referred to Gaucher and presented the cost-effectiveness of ERT compared to supportive care in the Netherlands (Table 1). Finally, one study was retrieved for LAL [16].

**Table 1:** Included studies

Author publication year	Country/ Perspective sponsored	Currency	Comparison	Treatment	Treatment Incremental Costs	Costs converted to PPP Euro 2022 Netherlands		Time Horizon	Discounting	Model Type	Utility scores (average)	LYG gained	QALY’s gained	ICER	Currency conversion to PPP 2022 Euro (Netherlands)
NCPE 2018	Societal perspective	2018 Euros	ERT compared to existing therapies	Sebelipase alfa Weight based dose Starting 0.35 mg/kg	N/R			Lifetime	NR	Cost-effectiveness state-transition model	NR	LAL-CL03: 67% 12-month survival LAL-CL02: N/A LAL-1-NH01 (Cohort): 0% > 12 months survival	NR	ICER: € 2,813,000/QALY (infantile cohort) € 2,701,000/QALY (paediatric adult cohort)	ICER: € 2,940,582/QALY (infantile cohort) 2,823,502/ QALY (paediatric adult cohort
Castro Jaramillo 2012	Colombia NHS perspective No funding	2012GBP	ERT compared to supportive therapy. (IOPD)	Alglucosidase 20 mg/kg/2 weeks	£ £557,653	€755,875.60		20 years	5%	Cost-effectiveness micro-simulation model	0.700	NR	5.07	(ICER): £ 109.991 per QALY earned	ICER 149,088 per QALY earned
Castro Jaramillo 2012	England NHS perspective No funding	201GBP	ERT compared to supportive therapy. (IOPD)	Alglucosidase 20 mg/kg/2 weeks	£ 1187.940	€1,610,203		20 years	5%	Cost-effectiveness patient-simulation model	0.700	NR	5.07	ICER £234.308 per QALY earned	ICER 317,594
Kanters et al. 2014	Netherlands Societal perspective funded by the Netherlands Organization for Health Research and Development	2009 Euros	ERT compared to supportive therapy (IOPD)	Alglucosidase 20 mg/kg/2 weeks	€1,9 million	€2,130,300		Lifetime	4% cost and 1,5% effect	Cost-effectiveness patient-simulation model	0.24–0.82 (0.62)	13.39	6,75	ICER: € 286.114 per QALY gained	320,794
Kanters et al. 2014	Netherlands Societal perspective funded by the Netherlands Organization for Health Research and Development	2009 Euros	ERT compared to Supportive therapy (IOPD)	Alglucosidase 40 mg/kg/weeks	€7,032,899	€7,885,363		Lifetime	4% cost and 1,5% effect	Cost-effectiveness patient-simulation model	0.24–0.82 (0.62)	13.39	6.75	ICER €1.043.868 per QALY gained	ICER €1,170,396 per QALY
Kanters et al. 2017	Netherlands Societal perspective funded by the Netherlands Organization for Health Research and Development	2014 Euros	ERT compared to Supportive therapy (LOPD)	Alglucosidase 20 mg/kg/2 weeks	Sc.1: €6.5 million Sc.2:€7.6 million	Sc 1: €7,117,241.Sc 2:€8,321,697		Lifetime	1,5% effects, 4.0% costs	Cost-effectiveness patient-simulation model	0,45	Scenario 1: 2.03 Scenario 2: 5.67	Scenario 1: 2.13 Scenario 2: 4.38	ICER (SCENARIO 1) €3.167.914 million per QALY gained SCENARIO 2 €1.774.390 million per QALY gained	ICER (SCENARIO 1)€3.468.739 per QALY gained Scenario 2 € 1.942.886 per QALY gained
Rombach et. Al. 2013	Netherlands Societal perspective Funded by a grant from the Ministry of Health	2009 Euros	ERT compared to Standard medical care (Fabry)	agalsidase alfa or beta	€ 2.420.956	€2,721,258		Lifetime	4% cost and 1,5% effect	Cost-effectiveness state-transition model	NR	NR	0.7 YFEOD:0.7	ICER €3.318.239 per YFEOD € 3.282.252 per QALY	ICER €3.729.842 per YFEOD ICER € 3.689.391 per QALY
Van Dussen et. Al., 2014	Netherlands Societal perspective Funded by the Dutch Top Institute Pharma project nr T6-208	2009 Euros	ERT compared to Standard medical care (Gaucher)	imiglucerase vial of 400UI	€ 1.206.933	€1,353,226		Lifetime	4% cost and 1,5% effect	Cost-effectiveness state-transition model	NR	NR	2.67 YFEOD: 5.8-	ICER €199,559 per YFEOD gained € 432,540 per QALY gained	ICER €223.747 per YFEOD ICER €484.968 per QALY gained

### Pompe Disease

#### Infant onset Pompe Disease (IOPD)

Kanters et. al. [6] used a patient simulation model to estimate the cost-effectiveness of ERT from a societal perspective in the Netherlands. Data on survival were obtained from international literature, as well as from a sample 20 classic-infantile patients in the Netherlands. The study employed a lifetime time horizon with 6-month time cycles. Discount rate on costs was 4% and 1.5% on effects. Health utilities were assumed equal in both treatment groups, due to the lack of data related to utility for patients receiving supportive care and were assessed using the Euroqol-5D (EQ-5D), every 6 months. The Dutch tariff A was also used. The analysis assumed that patients received 40 mg/kg/week (base case scenario) and 20 mg/kg/2 weeks of alglucosidase compared to supportive care alone. The results showed that the incremental cost (ICER) per QALY for the base case scenario was € 1.043.868, whereas the cost per life year gained (LYG) was 0.5 million euro. Patients gained 13.79 LY years on ERT, whereas the life expectancy on the ST group was marginal, leading to an incremental 13.39 LYG. The incremental QALYs gain was estimated at 6.75 (0.24 for the ST group versus 7 for the ERT group.) The total cost per patients on ERT was € 7,032,899 while the corresponding for the ST group was € 32,871. The cost of the product dominated the sensitivity analysis.

Castro-Jaramilo [5] created two deterministic Markov models from a health system’s perspective, to assess the cost-effectiveness of ERT for alglucosidase in infants in England and Colombia. England is a high-income country with a taxation-based health single payer health care system, while Colombia is a middle-income country with a health insurance scheme up consisting of a social security sector and a private sector. They proceeded with the creation of an economic model with a 20-year horizon. The efficacy data were synthesized from a pool of eight studies, to estimate the cost-effectiveness of ERT compared to supportive care, while the main comparative reviews of historic cohorts came from a 2003 Dutch study. [17]. The costs were set from the NHS perspective in UK and through payers in Colombia. Health utilities data were calculated using EQ-5D. A 5% discount rate was used on costs and effects. Authors estimated that the ICER per QALY for ERT was £234,307 for England and £109,991 for Colombia, compared to standard supportive/palliative care. The HR-QoL improved in the ERT group and was calculated at 0.7 while for the ST group, the HR-QoL was 0.388 for both models. The incremental QALY was estimated at 5.07, over the 20-year study horizon. Alglucosidase is administered every 2 weeks to patients with IOPD (20 mg / kg). Results revealed that the ICER per QALY obtained was £234,308 (£ 1,187,940 additional costs and 5.07 QALYs) for England and £ 109,991 (£ 557,653 additional costs and 5.07 additional QALYs) for Colombia. In this model, the total annual cost per patient (average weight, 10 kg), including ERT costs, was £ 194,342 in England and £97,963 in Colombia. The use of pediatric intensive care unit (£ 148,200 and £ 49,351 per patient for England and Colombia, respectively) dominated the cost-decomposition in the sensitivity analysis. ERT itself comprised the second largest cost center (£38,324 and £41,678 per patient for England and Colombia, respectively).

#### Late onset Pompe disease

Kanters et. al., [14] used a patient-level simulation model to assess cost-effectiveness of ERT for adult patients in Netherlands. The survival probabilities were estimated from an international observational dataset (The international Pompe Association (IPA)/ Erasmus MC Pompe Survey). The health utilities were assessed in Dutch patients using the Euroqol-5D (EQ-5D), completed by parents of patients every six months. Costs were measured from a societal perspective. Two scenarios were modeled: (1) a worst-case scenario with no extrapolation of the ERT-related survival gain, beyond the observed period (i.e., from 10 years onwards); and (2) a best-case scenario with lifetime extrapolation of the ERT-induced survival gain. The effects were expressed in QALYs. Costs were discounted at 4.0% and effects at 1.5%. Results showed that the discounted lifetime incremental costs reported for a person with LOPD, based on a dosage 20 mg /kg biweekly comprised, in principle, by drug costs. In the first scenario the incremental costs were estimated at €6.5 million and in the second scenario at € 7.6 million. For the cost-effectiveness of ERT in LOPD, the data were derived from a simulation model that uses Dutch patient-level data from a social point of view during life [14]. This study modeled two scenarios: an approach that did not yield results after the observed period (scenario 1) and an approach in which ERT's impact on survival extends beyond the observation period (scenario 2). In this analysis, ICERs were lower in Scenario 2 (€ 1.4 million per year of life and € 1.8 million per additional QALY versus € 3.4 million per year of life and € 3.2 million per additional QALY for Scenario 1). The utilities were increased by 0.03 in both scenarios (0.42 for the ST group and 0.45 for the ERT group). The life expectancy increased by 1.89 in the 1st scenario (16.33 years for the ST group versus 18.21 years for the ERT group), and by 5.44 in the 2nd scenario (16.42 years for the ST group versus 21.84 years for the ERT group). The difference in QALYs in the first scenario was 2.04 (10.53 for the ST group and 12.57 for the ERT group) and for the second scenario was 4.26 years (10.60 and 14.85 for the ST group and the ERT respectively).

### Fabry disease

Rombach et. al., [9] performed a cost-effectiveness analysis using a life-time state-transition model to evaluate the costs and effects of intervention with ERT (either agalsidase alfa or agalsidase beta) compared to standard medical care for Fabry disease in Netherlands. Transition probabilities, effectiveness data and costs were derived from retrospective data and prospective follow-up of the Dutch study cohort consisting of males and females aged 5–78 years. [9]. The main outcome measures were years free of end organ damage (YFEOD) (renal and cardiac complications), QALYs and costs. ERT treatment was performed with either agalsidase alpha or agalsidase beta, which was based on authors discrepancy. The average annual cost of ERT per patient was calculated based on the needs of a 70-kg patient (price list for 2009: € 200,503 for agalsidase alpha and € 199,452 for agalsidase beta) at € 2,504,727 (with a 4% discount) per year. The non-discounted life-time costs of ERT patients accrued to € 9.9 million during the lifetime course of patients, dominated by the acquisition costs of the product, compared to € 0.271 million on patients receiving only basic medical care. The results demonstrated that over a lifetime of 70 years, an untreated Fabry patient would have attained 55.0 years without end-stage organ damage and 48.6 QALYs. Initiation of ERT in a symptomatic patient leads to an undiscounted incremental gain of 1.5 for YFEOD and 1.6 QALY (both 0.7 discounted). Consequently, the additional costs per additional YFEOD and the additional costs per additional QALY gained ranged from € 5.5 to € 7.5 million. The ICER based on years free of end organ damage equals to 6.6 million euros, while the additional cost per QALY respectively to 6.1 million euros (undiscounted). Assuming a discount of 1.5% for effects and 4% for the costs, the ICER per YFEOD reduced to 3.3 million euros, while the additional cost per QALY decreased respectively to 3.28 million euro.

### Gaucher disease

Van Dussen et. al., [15] performed a cost- effectiveness analysis using a Markov state-transition model of the disease’s natural course to evaluate the cost-effectiveness of enzyme replacement therapy compared to standard medical care without ERT in the Dutch cohort of patients with type 1 Gaucher disease. The mode was developed with data from the Dutch Gaucher registry. Transition probabilities, costs and effectiveness data were derived from retrospective data and prospective follow-up of the Dutch study cohort. ERT treatment (starting at symptomatic stage) resulted in an average cost of living of € 5,716,473 (cost difference compared to patients who did not receive ERT, € 5,544,693). According to the baseline scenario, type 1 Gaucher patients treated with ERT had 61.7 YFEOD (discounted 37.77) and 62.13 QALYs (discounted 37.33). After discount ERT patients generated €1,206,933 during their lifetime. According to van Dussen et. al., the administration of ERT treatment to a symptomatic patient increased YFEODs by 12.8 years (discounted 5.80), while the number of QALYs acquired increased by 6.27 (discounted 2.67), i.e., 61.7 YFEODs and 62.13 QALYs. The incremental cost-effectiveness ratio of ERT against standard care was estimated €434,416 per YFEOD (discounted €199,559) and €884.994 per QALY (discounted €432,540).

### Lysosomal acid lipase deficiency

The NCPE assessed the use of sebelipase for LAL deficiency. The LAL deficiency in infantile patients causes the Wolman disease [16]. The survival rates were estimated based on the cohort of VITAL study patients, which survived over the first year (56% survived > 24 months) compared to a historical comparison study (NH01) [18]. The primary health outcome was QALY, and costs were estimated using national DRGs. The utility values were derived from the UK EQ-5D population norms. No utility decrements were included for the infantile cohort. Results showed that the ICER per QALY for the base case was €2,284,000/QALY for the infantile cohort. The probability of being cost-effective at a willingness-to-pay threshold of €45,000/QALY was 0% in both models (infantile and paediatric). The NCPE incorporated utility decrements, alternative transition probabilities and treatment effectiveness estimates on cost effectiveness resulting an ICER up to €2,813,00/QALY.

LAL deficiency is expressed in adults as the Cholesteryl Ester storage disease. The economic model was based on fibrosis progression rate and the data were mined from the ARISE study (multicenter, randomized, double blind, placebo controlled) [19]. Nevertheless, it should be underlined that no data from trials regarding liver disease progression between BSC and sebelipase alfa in the pediatric adult patients where included. Authors used the reported health state cost from a study about Hepatitis C [20]. Utility values were derived from the UK EQ-5D population norms while decrements were from Crossan et.al., [21]. The incremental cost per QALY (incremental cost-effectiveness ratio (ICER)) for the applicant’s base case was €1,790,000/QALY for the pediatric adult cohort. The NCPE incorporated utility decrements, alternative transition probabilities and treatment effectiveness estimates on cost effectiveness perpetuated to an ICER of €2,701,000/QALY.

Moreover, authors estimated that the projected cumulative gross drug budget impact over the first five years, with the addition of administration costs, was €23.5 million.

## Quality of the studies

The quality of economic evaluations comprises a crucial factor. We assessed the methodological quality with the Quality of Health Financial Studies (QHES) (Table 2). QHES is a validated tool consisting of 16 weighted points..QHES offers value by elaborating a qualitative analysis of the results of assessment of individual items. The underlying assumption is that higher quality studies will lead to better decision-making framework, by compounding bias and misuse Overall, the included studies demonstrate a favorable methodological quality, which bolsters their potential contribution in the decision-making process [22].

**Table 2:** Quality of Health Financial Studies (QHES)

	Castro-Jaramillo (2012)	Kanters et al. (2014)	Kanters et al. (2017)	Rombach S.M. et.al., (2013)	Van Dussen L. et.al., (2014)	NCPE
Was the study objective presented in a clear, specific, and measurable manner?	7	7	7	7	7	7
Were the perspective of the analysis (societal, third-party payer, etc.) and reason for its selection stated	4	4	4	4	4	
Were variable estimates used in the analysis from the best available source (i.e. Randomized Control Trial –Best, Expert Opinion –Worst)?	8	8	8	8	8	8
If estimates came from a subgroup analysis, were the groups prespecified at the beginning of the study?	1	1	1	1	1	1
5) Was uncertainty handled by: 1) statistical analysis to address random events; 2) sensitivity analysis to cover a range of assumptions?	9	9	9	9	9	9
Was incremental analysis performed between alternatives for resources and costs?	6	6	6	6	6	6
Was the methodology for data abstraction (including the value of health states and other benefits) stated?	5	5		5	5	
Did the analytic horizon allow time for all relevant and important outcomes? Were benefits and cost that went beyond one year discounted and a justification given for the discount rate?	7	7	7	7	7	7
Was the measurement of costs appropriate and the methodology for the estimation of quantities and unit costs clearly described?	8	8		8	8	8
Were the primary outcome measure(s) for the economic evaluation clearly stated and were the major short-term, long-term, and negative outcomes included?	6	6	6	6	6	6
Were the health outcomes measures/scales valid and reliable? If previously tested, valid and reliable measures were not available, was justification given for the measures/scale and reliable measures were not available, was justification given for the measures/scale used?	7	7	7	7	7	7
Was the economic model (including structure), study methods and analysis, and the components of the numerator and denominator displayed in a clear transparent manner?	8	8	8	8	8	
Were the choice of economic model, main assumptions and limitations of the study stated and justified?	7	7	7	7	7	
Did the author(s) explicitly discuss direction and magnitude of potential biases?					6	
Were the conclusion/ recommendations of the study justified and based on the study results?	8	8	8	8	8	8
Was there a statement disclosing the source of funding for the study?			3	3	3	
Total score	91	91	81	94	100	67

## Discussion

The context of ERT entails an array of products for a cluster of orphan diseases and—almost unanimously—these agents comprise the unique available therapeutic modality. This leaves payers facing mounting costs for this patient cohort, with slim feasible alternative strategies. This is even more relevant in the face of the pandemic, since health systems, on a global level, strive to sustain both the operational efficiency and fiscal sustainability [23].

A long-simmering public debate regarding the reimbursement of orphan medicines has reached a stalemate and while the reported ICERs greatly exceed even the highest thresholds across included countries, ERT are routinely reimbursed in many countries, embroiling payers to a trivial dilemma. Such decisions are not stand-alone: the ramifications extend and permeate other orphan diseases as well and this may as well preoccupy decision-makers and escalate to a rule of thumb, for other rare health conditions.

The economic evaluation of sebelipase alfa for lysosomal acid lipase deficiency (LAL-D.) reflects the impediments that engulf the reimbursement of ERT [17]. The ICER per QALY for the infantile patients was estimated at €2,813,000/QALY and €2,701,000/QALY for the adult population. The probability of the treatment being cost-effective at a willingness-to-pay threshold €45,000/QALY is non-existent, while the budget impact is substantial, at €23.5 million over a five-year horizon. Therefore, NCPE recommended against the reimbursement of sebelipase alfa, either for infantile or pediatric adult patients, even in the backdrop of the demonstrated additional benefit in survival for infantile patients and evidence of clinical improvement in pediatric adult patients. [18] [19] [24] [25]

Before we unfold the hot topic of affordability of ERT, which dwarfs all other costs centers of all included economic evaluations, we should deliberate the differences between the results of economic evaluations in our study. Apart from the local cost differences across countries, the discounting factors comprise a source of variability. Most of the economic models employed a lifetime scenario, which spirals to significant differences pertinent to the selected discount rate.

The ERT cost dominates the economic evaluation and solidly all economic evaluations underpin the impact of pharmaceutical cost in the cost matrix. Consequently, any reduction in ERT price would substantially reduce overall costs and deliver favorable ICER. Nevertheless, it should mandate steep reductions to render them as cost-effective.

We also noticed the oxymoron of higher prices in country with lower GDP, as in the case of average ERT cost per case in Colombia, which exceeds the corresponding cost in England. It should also be underlined that the low demand for ERT due to the rarity of the diseases has a direct effect on lowering the importance of providing ERT to the national healthcare budget even if the cost per QALY acquired is favorable.

Significant differences were noted across the retrieved LSD studies, a finding that rules out a class effect in the ERT category. The cost per QALY for ERT on IOPD disease was estimated to be 1 million euros and the cost per LYG was 0.5 million euros which extends well above the conventional Willingness-to-Pay (WTP) threshold values. Nevertheless, the therapy exerted a substantial effect on the life expectancy of the patients, which increased noticeably (13.8 years whereas the group who received the supportive therapy did not exceed the 0.40 years of life).

For LOPD the incremental cost per life year gained was 1.4 million euros and the incremental cost per QALY ratio was estimated at 1.8 million euros. QALYs were increased by 6.75 years with ERT for IOPD and for LOPD it ranged between 2.04 and 4.26 incremental QALYs. The utilities were increased by 0.03 (0.42 for the ST group and 0.45 for the ERT group).

The incremental cost per QALY gained with ERT for Fabry disease is estimated at 3.3 million euros [9]. The therapeutic effect of ERT in symptomatic patient with Fabry disease is marginal, while it has limited effect on the quality of life and progression to end organ damage. This lack of potency spirals to the non-cost-effectiveness outcome. With ERT the QALYs increased from 48.6 to 50.2 (difference 1.6) and the YFEOD increased by similar amount (from 55.0 to 56.5) leaving the affordability of ERT of Fabry disease at stake.

ERT increases substantially the QALYs and the YFEOD for Gaucher disease and therefore, it is anticipated that in the long term, can effectively improve quality of life. Over an 85-year lifetime, ERT increases QALYs from 55.86 to 62.13 and the YFEOD increase from 48.9 to 61,7 for a Gaucher patient. While the ICER was lower, compared to the other agents, it still exceeded £200,000 per QALY. Nevertheless, we should underline a flaw of this publication. The investigators assumed equal health utilities and costs (excluding ERT medication) for treated and untreated patients during the same disease states [26]. The sensitivity analyses of retrieved studies suggest that the cost of ERT dominates every sensitivity analysis scenario and a reduction in prices wielded a steep beneficial effect on ICER. The cost reduction can be seemingly achieved by lowering the dose of ERT, however a temptation to lower the dose and thus the cost, may exert a negative effect on the therapeutic outcome, which in return neutralizes any net benefit on ICER.

A vital part of ERT efficiency is nested in the selection process of patients, which should incorporate parameters such as age and stage of the disease, which in return can maximize the beneficial outcome. As discussed, ERT was associated with better results in children with IOPD compared to adult patients with LOPD. Additionally, Rombach et al. [9] pointed out potential gender difference, in the sense that ERT showed slightly better results for males. Incremental lifetime medical costs for males were €9,343,028 and for females were €9,789,106 (undiscounted). There was also a difference in the effects of ERT. Males gained 1.6 YFEOD with ERT (55.1 with ERT than 53.5 with no ERT) while females gained 1.3 (58.2 with ERT than 56.9 with no ERT). QALYs with ERT assessed to be 49.5 versus 47.8 with no ERT for males while for females QALYs with ERT assessed to be 51.1 versus 49.7 with no ERT. However, this can be overlooked because females tent to live longer.

Currently several authors advocate the potential exception of orphan drugs from cost-effectiveness assessment [27]. The most convoluted question is whether society is willing to pay a premium because of the rarity of a disease. Critics expostulate with an unconditional reimbursement since this will jeopardize sufficient funding in other healthcare segments. Nevertheless, the issue is more complicated and merits further discussion. Primarily, non-reimbursement on grounds of non-cost-effectiveness would be fiercely opposed both at a political and a social level. Indicatively the appraisal framework in the UK endorses the use of lower evidential standards for orphan drugs. One solution is to scrutinize further the selection of patients and distinguish which patients are going to benefit from it, exploring the sensitivity analysis as well. Such approaches are usually already embedded in the clinical guidelines as well.

Primarily, we should answer a frequently raised question following such economic reviews, whether it make sense to perform more economic evaluations of ERT. The prerequisite for a lower WTP, apart from the prices, implies vast gains in QALY, which are virtually beyond the capacity sphere of these agents. Indicatively, Wyatt et. Al. [28] argued that if each year, ERT delivered an additional 4.2–4.8 QALYs in a pool of 128 adults it would be cost-effective considering a theoretical ICER of £30.000. ERT for Fabry patients would necessitate at least 3.6 discounted QALYs for an adult patient with Fabry disease, under a £30,000 WTP threshold. Clearly, both approaches are unfeasible out of context.

Therefore, we should contemplate whether it is socially justifiable to relax the WTP thresholds and focus on the budget impact of these products, coupled with a thorough selection of patients that will gain the most benefit.

The majority of the countries do not explicitly define a WTP. As per WHO guidance, the WTP can be set in accordance to the financial state of each country. The WHO recommends a threshold between three and five times the national annual gross domestic product (GDP) per capita. Notably modalities that yield a threshold less than once the national annual GDP per capita are considered highly cost-effective [29].

Cost-effectiveness analysis remains a useful valuable tool in the reimbursement decision process. Nevertheless, it is affected using unweighted QALYs gained. This conflicts with an approach usually found in other parts of social life, the rule of rescue, which refers to the practice of spending inordinate amount of money to save people from life-threatening situations, albeit when performing non-essential activities as sailing and climbing. By extrapolating this in health, this prioritizes people who are in worst position in the sense of disease severity and urgency of needed intervention, will be treated accordingly and their abandonment is ruled out. ICER conveys the opportunity cost, which infers that the quest is to maximize the alternative gains elsewhere in the system. This assumes that all QALYs are equal, an issue which drawn significant criticism. This aligns with the EU norm, which demands that patients should have the same quality of treatment. The issue of orphan disease is further intricated by the interlace of budget impact and cost-effectiveness. In the case of orphan disease, cost-effectiveness does not report size of cost or benefit. Therefore, in certain cases, the unfavorable ICERs of orphan medicines do not consider that the industry recoup high and fixed costs from a small pool of patients. This implies discrimination against these individuals. Finally, cost-effectiveness implies that ‘if a person remains miserable or painfully ill, her deprivation is not obliterated or remedied or overpowered simply by making someone else happier or healthier [29].

## Limitations of this study

Significant limitations of this ERT evaluation study include the small number of studies that have been published that provide data on the cost and cost-effectiveness of ERT. At the same time, these studies consisted of a very small number of patients, and several were non-random, however, the rarity of these diseases allows a small number of patients in the studies as it is representative of the population. In addition, in several studies, there were data on cost but not on cost-effectiveness and thus a comprehensive analysis was not possible. Finally, the ERT dose and the frequency of ERT administration vary among countries, which may affect the transferability of these results to other countries. This parameter is important as the ERT regimen will affect the cost of treatment and may affect the course of the disease. Nevertheless, relevant comparisons can be made between data from different countries.

## Recommendations for further research

It is anticipated that data of gene therapies will be available, following the preliminary data of AVR-RD-01 for Fabry and Gaucher disease. This will transform the disease management; however, it will augment fiscal pressures. The case of Zynteglo, a promising gene treatment for thalassemia, which comes at a burgeoning cost of 2 million is a representative case. Despite demonstrating significant benefit, its soaring price left health system balked and currently the product is not available in EU countries.

In addition, further research could help clarify the many uncertainties that emerged, along with economic data especially for diseases for which data are not available, such as mucosal polysaccharides (MPS) and immunodeficiency (SCID) ADA enzyme deficiency. It is highly unlikely that, regardless of the findings of any research, ICER could be reduced to the level required to comprise ERT eligible for reimbursement under the conventional pathway.

Therefore, more clinical data regarding the life expectancy after ERT, survival rates and quality of life after long term treatment should compounds the medical uncertainty. This should be coupled with decision regarding reimbursement, either in the form of relaxing WTP, focusing on BIA, rather than unequivocally on CEA and repurposing the principles of solidarity.

## Data Availability

The datasets used and/or analyzed during the current study are available from the corresponding author on reasonable request.
