# Correction to: A systematic review of economic evaluations of enzyme replacement therapy in Lysosomal storage diseases

**DOI:** 10.1186/s12962-022-00392-x

**Published:** 2022-12-06

**Authors:** Eleni Ioanna Katsigianni, Panagiotis Petrou

**Affiliations:** Pharmacy School, Department of Life & Health Sciences, School of Sciences and Engineering, University of Nicosia, 2417 Nicosia, Cyprus

## Correction to: Cost Effectiveness and Resource Allocation 20:51 (2022) 10.1186/s12962-022-00369-w

Following publication of the original article [1], the authors flagged that the corrections they requested during the proofing of their article had not been implemented correctly. The article has since been updated to implement the requested corrections, and the requested corrections can be seen in the file attached to this erratum.

The publisher thanks you for reading and apologizes for any inconvenience caused.

## Supplementary Information

**Additional file 1.** Correction requested for proof https://doi.org/10.1186/s12962-022-00369-w.

## References

[1] Katsigianni EI, Petrou P (2022). A systematic review of economic evaluations of enzyme replacement therapy in Lysosomal storage diseases. Cost Eff Resour Alloc.

